# Effects of *Achyranthes bidentata* Polysaccharides on Intestinal Morphology, Immune Response, and Gut Microbiome in Yellow Broiler Chickens Challenged with *Escherichia coli* K88

**DOI:** 10.3390/polym10111233

**Published:** 2018-11-07

**Authors:** Zhuying Liu, Xiaolong Wang, Shuqi Ou, Muhammed Adebayo Arowolo, De-Xing Hou, Jianhua He

**Affiliations:** 1 College of Animal Science and Technology, Hunan Agricultural University, Changsha 410128, Hunan, China; liuzhuying@gmail.com (Z.L.); wangxiaolong1982@gmail.com (X.W.); 15574942599@163.com (S.O.); mbayor88@gmail.com (M.A.A.); 2 The United Graduate School of Agricultural Science, Kagoshima University, Kagoshima 890-0065, Japan; hou@chem.agri.kagoshima-u.ac.jp

**Keywords:** *Achyranthes bidentata* polysaccharides, immune response, intestinal mucosal morphology, cecal microbiota, *Escherichia coli* K88

## Abstract

The present study was conducted to investigate the effects of dietary *Achyranthes bidentata* polysaccharide (ABPS) supplementation on performance, immune response, intestinal mucosal morphology, and gut microbiome in yellow-feathered broilers challenged with *Escherichia coli* K88. A 2 × 2 factorial design was used for the trial. Two hundred and forty one-day-old female broilers were randomly assigned to four treatments: (1) negative-control broilers were fed by a basal diet and saline (NG); (2) positive-control broilers were fed by a basal diet and orally challenged with 10^8^ CFU *E. coli* K88 (CNG); (3) ABP group broilers were fed by a basal diet containing ABPS (500 mg/kg of feed) and saline; (4) CABP group broilers were fed by a basal diet containing ABPS (500 mg/kg of feed) and orally challenged with 10^8^ CFU *E. coli* K88. Growth performance, serum biochemical indexes, immune responses, intestinal mucosal morphology, and cecal microbial community structure were evaluated. The ABP group had greatest body weight (BW), average daily body weight gain (ADG), and intestinal villus height compared to other treatments (*p* < 0.05). The CABP group had a higher villus height/crypt depth ratio (V/C) compared with other treatments (*p* < 0.05). The expression levels of NF-κB were lower in the ABP group. The CNG group had higher *Escherichia coli* and *Enterococcus* contents in cecal samples compared to other treatments (*p* < 0.05). Serum glucose, uric acid, TNF-α, and Secretory Immunoglobulin A (S-IgA) levels were higher in broilers challenged with *E. coli* (*p* < 0.001) than that with saline. Broilers challenged with *E. coli* had reduced taxa richness in the cecal samples. Sequencing of 16S rRNA genes in cecal samples revealed that a lower proportion of Firmicutes and a higher proportion of Proteobacteria were detected in the broilers challenged with *E. coli*. Compared with the controls, dietary ABPS supplementation increased serum total protein, albumin, and S-IgA levels, but decreased serum glucose, uric acid, and TNF-α levels in broilers (*p* < 0.05). Diet supplemented with ABPS increased the Firmicutes/Bacteroidetes ratio and the abundance of *Ruminococcaceae* and *Lachnospiraceae*, and altered cecal microbiota community structure. These results suggest that ABPS can promote growth performance and improve intestinal morphology and microbiota community structure in broilers challenged with *E. coli* K88*.*

## 1. Introduction

The principal pathogenic bacteria associated with poultry causing foodborne illnesses are *Campylobacter*, *Salmonella*, and *Escherichia coli* [1]. *Escherichia coli* can cause an enteric disease known as colibacillosis in chickens, which in turn results in a high mortality rate and huge economic loss in the poultry industry [2,3]. Although colibacillosis can be prevented or treated by the use of antibiotics, there exist controversies regarding the prophylactic use of antibiotics in livestock production throughout the world. Therefore, alternative strategies have been actively investigated to replace these synthetic antimicrobial agents. Many studies have been published about different additives’ effects on broiler chicken performance and immune response when challenged with *Escherichia coli* K88, such as prebiotics, probiotic, short-chain fatty acids, and essential oils, but little is known about polysaccharide additives [4,5,6,7]. These additives promote growth performance by increasing the average daily feed intake and improving immune function and intestinal villi development, but there has been very little research on the effects on the intestinal microbial community structure and balance of the host [8,9].

Polysaccharides extracted from traditional Chinese medicines have been regarded as one of the alternatives, and a growing number of studies have shown that the polysaccharides have beneficial effects in promoting the growth performance and health of livestock animals [10]. *Achyranthes bidentata* polysaccharides (ABPS) are polysaccharides extracted from the Chinese herb *Achyranthes bidentata.* Several in vitro and in vivo studies have revealed the immunostimulating and anti-inflammatory effects of ABPS [11,12]. ABPS was also shown to enhance immune responses, increase feed intake, and promote growth when incorporated into swine diets, but little research has been carried out with poultry [13,14]. In our laboratory, we have observed a salutary influence of ABPS supplementation on pigs [15,16,17]. Moreover, our preliminary data showed that ABPS supplemented at the rate of 500 mg/kg of diet increased lymphocyte proliferation and improved immunity in yellow broiler chickens [18]. Based on these results, the present study was designed to investigate the effects of ABPS supplemented at the 500 mg/kg level of inclusion on growth performance, immune function, and gut microbiome community structure in yellow-feathered broiler chickens challenged with *Escherichia coli* K88.

## 2. Materials and Methods

### 2.1. Birds, Diets, and Experimental Design 

#### 2.1.1. Preparation of ABPS and *E. coli* K88

ABPS was extracted and prepared according to the procedure described by Chen et al. [15]. The extract was comprised of *D*-mannose and *D*-glucose in a molar ratio of 8:1 (HPLC analysis, Shanghai Institute of Chemical Physics, Chinese Academy of Sciences, Shanghai, China), and the final product was 95% ABPS as measured by vitriol anthrone using anhydrous glucose as standard control. The relative molecular mass of the ABPS was about 1300–1400 D as determined by the gel filtration method. The final extract was used for this trial [19].

The *E. coli* K88 strain was originally obtained from the College of Animal Sciences and Technology, Hunan Agricultural University, Changsha, China. The strain was inoculated in Luria Bertani broth for 24 h and cultured at 37 °C with shaking (120 rpm). To determine the appropriate incubation time, a growth curve was constructed to establish the density of *E. coli* K88 required to reach the target gavage dosage of approximately 2 × 10^8^ CFU/mL. 

#### 2.1.2. Animals 

All the experimental procedures were approved by the Institutional Animal Care and Use Committee of Hunan Agricultural University. In total, 240 1-day old female yellow-feathered broiler chickens (average BW = 30.4 g) were obtained from Hunan Wenshi Poultry Industrial Development Company, China. They were inspected upon arrival to ensure all chicks were free from any deformity and early signs of disease. Healthy chicks were randomly assigned to 4 treatments (with 6 replicates of 10 chicks each). Each cage had raised wire floors and contained a self-feeder and waterer. The room temperatures were adjusted to 32 °C in the first week, 30 °C in the second week, 28 °C in the third week, and then 25 °C to the end of the study.

#### 2.1.3. Diets and Experimental Design

The diets used in this study were formulated based on the nutrient requirements of yellow-feathered broiler chickens (China, NY/T 33-2014) and the NRC (1994) (Table 1). All groups were fed the basal diet without growth promoters nor anticoccidia for the first 14 days, but were vaccinated against coccidiosis at placement. A 2 × 2 factorial design was used for the current study. The treatments were as follows: the negative treatment group received a basal diet and saline (NG); the positive treatment group challenged by *E. coli* K88 received a basal diet (CNG); the third group received a basal diet supplemented with ABPS (500 mg/ kg of feed) and saline (ABP); and the fourth group challenged by *E. coli* K88 received a basal diet supplemented with ABPS (500 mg/kg of feed) (CABP). *E. coli* K88 at the concentration of 2 × 10^8^ CFU/mL of sterile saline was administered to the challenged groups at days 14, 15, and 16. This was achieved by dropping 0.5 mL of the solution at the back of the oral cavity with the aid of a sterile syringe attached to a polyethylene tube, whereas the unchallenged groups were gavaged only with 0.5 mL of sterile saline. The *E. coli* K88-challenged and the unchallenged groups were kept in two separated rooms to avoid cross-contamination. Traditionally, yellow broiler chickens are always divided into early phase (1–28 day) and late phase (29–56 day); the experimental on the effects of ABPS supplementation was focused on the early phase, thus the experiment lasted 28 days.

### 2.2. Data and Sampling Collection

#### 2.2.1. Growth Performance 

Individual bodyweight of the birds was measured at 0 and 28 days old. Similarly, mortality and feed intake per cage were recorded daily. The growth performance of broilers was calculated by the average daily feed intake (ADFI), average daily body weight gain (ADG), and feed/gain ratio (FCR), which were calculated according to the data from each cage.

#### 2.2.2. Blood Samples

At day 28, six birds were randomly selected from each group (1 bird from each cage) and were fasted for 12 h before being sacrificed. Five milliliters of blood sample per bird were collected by venipuncture of the wing vein. Serum were obtained by centrifugation at 3000× *g* for 10 min at 4 °C and stored at −20 °C until serum biochemical analyses. Serum biochemical indices (glucose, total protein, albumin, globulin, and uric acid) were analyzed using a BS-200 automatic blood biochemical analyzer (Mindray, Shengzhen, China).

#### 2.2.3. Fecal and Jejunal Mucosa Samples

The birds were euthanized, then approximately 1 g of cecal feces was immediately isolated from the middle of the cecum. The cecal samples were collected into 1.5 mL Eppendorf tubes and quickly frozen in liquid nitrogen while being transported to the laboratory. A 5-cm segment of the intestine was cut from the midpoint of the jejunum. The tissue samples were lightly flushed with ice-cold physiological saline (154 mmol/L). Approximately 1 g jejunal mucosa was immediately isolated from the middle of the jejunum with sterilized surgical scissors. The jejunal mucosa samples were collected into 1.5 mL Eppendorf tubes and quickly frozen in liquid nitrogen while being transported to the laboratory. All of the samples were stored at −80 °C until further processing.

#### 2.2.4. Duodenum, Jejunum, and Ileum Samples

The small intestine was promptly moved out and divided into 3 parts: the duodenum (from the pylorus to the distal point of entry of the bile ducts), jejunum (Meckel’s diverticulum marked the end point of the jejunum), and ileum (the ileocecal junction marked the end of the ileum). A 1-cm segment of the intestine was cut from the midpoint of each of the duodenum, jejunum, and ileum. These intestinal tissue samples were lightly flushed with physiological saline (154 mmol/L), blotted dry with filter paper, and fixed into a 4% paraformaldehyde fixing solution at 4 °C until further analysis of intestinal mucosal morphology [20].

### 2.3. Mucosal Cytokines and S-IgA

Jejunal mucosa (0.5 g per sample) was weighted, diluted with 9 mL of 0.9% saline, homogenized at 4 °C, and then centrifuged at 6000× *g* for 15 min at 4 °C. The supernatant was harvested into 1.5 mL Eppendorf tubes. The concentrations of tumor necrosis factor-a (TNF-α), IL-4, and secreted IgA (S-IgA) were analyzed by ELISA (Lan Bao Biological Technology Co. Ltd., Hangzhou, China).

### 2.4. Measurement of Intestinal Mucosal Morphology

One-centimeter intestinal tissue samples of the duodenum, jejunum, and ileum were embedded in paraffin. A microtome (RM-2235, Leica microsystems AG., Hessen, Germany) was used to make 5- or 6-μm slices that were mounted in glass slides and subsequently stained with hematoxylin and eosin (HE staining). Villus height (from the tip of the villus to the villus crypt junction) and crypt depth (from villus crypt junction to the base of the crypt) were measured under an Olympus Van-Ox S microscope (Opelco, Washington, DC, USA) using an image analysis software (Image-Pro, Media Cybernetics, Inc., Silver Springs, MD, USA). Six sections were taken from each intestine, and the height of the ten largest villi and the deepest crypt depth were selected for each section. From this, the villus height/crypt depth (V/C) values were calculated [20,21].

### 2.5. Jejunum Immunohistochemistry (IHC)

Measurement of IHC was as described previously [22]. Briefly, 1 cm of jejunum tissue (4% PFA-fixed and paraffin-embedded) was cut into six sections and mounted on slides. For IHC, slides were placed in an oven with 56 °C drying heat for 30 min for deparaffinization, then washed in alcohol and xylol solutions. Slides were placed in citrate buffer in a histoprocessor T/T MEGA instrument at 98 °C for 5 min for antigen retrieval. Then, they were washed in phosphate-buffered saline (PBS). For blocking the endogenous peroxidase, sections were incubated in 3% H_2_O_2_ for 10 min. Slides were placed in distilled water and PBS, then incubated with primary antibody (anti-NF-κB pAb ab16502 Abcam, diluted, 1:50 and MAPK(ERK1/2) anti-ERK1/2 #4696 CST, diluted, 1:1000) for 1 h and washed with PBS. Then, the slides were incubated with a second antibody, IgG antibody horseradish peroxidase (HRP), for 30 min and washed with PBS. Application of the diaminobenzidine hydrochloride chromogen for 10 min and washing with tap water were then performed. Only brown-colored staining was considered positive. All of slides were observed by light microscopy at 400× magnification, the selected field for counting being randomly chosen. Mean optical density of NF-κB and ERK1/2 in jejunum sections were analyzed using Image-Pro Plus 6.0 software (Media Cybernetics Inc., Bethesda, MD, USA).

### 2.6. Cecal Microbiota Analysis

DNA was extracted from cecal samples (one bird in each of the duplicate cages) using a Stool DNA Isolation Kit (Tiangen Biotech Co., Ltd., Beijing, China). The V3–V4 hypervariable region of the bacterial 16S rRNA gene were amplified by PCR, where the forward primer was 338F: 5′-ACTCCTACGGGAGGCAGCAG-3′ and the reverse primer was 806R: 5′-GGAC-TACHVGGGTWTCTAAT-3′. For each cecal sample, a 10-digit barcode sequence was added to the 5’ end of the forward and reverse primers (provided by Allwegene Company, Beijing, China). The volume of PCR reaction was 30 μL, containing 15 μL of Phusion^®^ High-Fidelity PCR Master Mix (New England Biolabs Inc., Beverly, MA, USA), 0.2 μM of forward and reverse primers, 10 ng of template DNA, and 13.8 μL double distilled H_2_O (ddH_2_O). Cycling parameters were 98 °C for 1 min, followed by 30 cycles at 98 °C for 10 s, 57 °C for 30 s, and 72 °C for 30 s, and a final extension at 72 °C for 10 min. PCR products were mixed in equidensity ratios and purified with the GeneJET Gel Extraction Kit (Thermo Fisher Scientific Inc., Schwerte, Germany), quantified using real-time PCR, and sequenced at Allwegene Company, Beijing. The sequences were clustered into operational taxonomic units (OTUs) at a similarity level of 97% to generate rarefaction curves and to calculate the richness and diversity indices. OTUs representing <0.005% of the population were removed and taxonomy was assigned by the Ribosomal Database Project (RDP) classifier. β-diversity was assessed by MANOVA and principal coordinate analysis. Significant differences in α-diversity and OTU counts between the different groups were determined by one-way and two-way ANOVA analysis followed by Duncan’s multiple comparison test using the SPSS (SPSS statistics 20) [23].

### 2.7. Statistical Analysis

Data were expressed as mean ± SEM and analyzed by one-way and two-way ANOVA for single-factor and two-factor designs, respectively, using SPSS (SPSS statistics 20). In the case of the two-way analysis, interactions between treatment factors were also assessed using the SPSS program. The mean differences among different treatments were separated by Duncan’s multiple range tests. A level of *p* < 0.05 was used as the criterion for statistical significance. The statistical analyses used in the assessment of microbial community structure (16S rRNA sequencing) was described in Section 2.6.

## 3. Results

### 3.1. Growth Performance

The interaction effect between *E. coli* challenge and ABPS on BW and ADG (*p* = 0.038) of the broilers across the group throughout the experimental period is shown in Table 2. The ADG and final BW of birds were increased significantly with the ABPS treatment compared to the other groups, while the lowest was observed with the CNG treatment. The birds’ FCR was increased in the CNG treatment and significantly different (*p* < 0.001) to the other groups. Diet supplemented with ABPS decreased FCR (*p* = 0.002). On the other hand, no significant difference was observed in the ADFI and mortality of yellow-feathered broiler chickens during *E. coli* challenge and ABPS treatment.

### 3.2. Serum Biochemistry Indices

The results of the serum biochemical indices are shown in Table 3. There was no interaction effect between *E. coli* challenge and ABPS for the serum biochemical indices that were observed throughout the trial. Supplementation of ABPS increased the concentrations of total protein (TP) and globulin (GLB) (*p* < 0.001) in both *E. coli*-challenged and unchallenged birds. The *E. coli* challenge had no significant effects on GLB concentration (*p* > 0.05) compared with birds in the NG group, but had the tendency to decrease the content. Dietary ABPS supplementation with no *E. coli* challenge (ABP) led to decreased levels of uric acid compared with other groups, especially the NG group. At the same time, *E. coli* challenge resulted in an increased level of uric acid (*p* < 0.001). Serum glucose was significantly lower (*p* < 0.001) in the groups supplemented with ABPS, whether challenged with *E. coli* or not. 

### 3.3. Immune Responses

**Figure 1:**
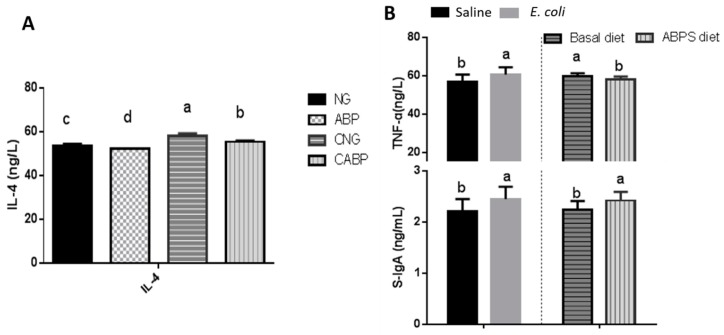
Effects of ABPS and *E. coli* challenge on mucosal cytokines and S-IgA in yellow-feathered broilers. (**A**) Concentration of IL-4 in jejunal mucosa; the interaction effect between *E. coli* challenge and ABPS was observed for IL-4 throughout the trial period. (**B**) Concentrations of TNF-α and S-IgA in jejunal mucosa; ABPS and *E. coli* treatments were significant (*p* < 0.05) for TNF-α and S-IgA. Values are means ± SE, *n* = 6. Different superscripted lowercase letters within each group mean the values differ significantly (*p* < 0.05). NG: basal diet; ABP: supplemented with 500 mg ABPS additive per kg of diet; CNG: basal diet group with *E. coli* challenge; CABP: ABP group with *E. coli* challenge.

As shown in Figure 1, an interaction effect between *E. coli* challenge and ABPS was observed for IL-4 throughout the trial period. The ABP group had the lowest concentration of IL-4 in jejunal mucosa compared with other treatments (*p* = 0.04). Birds in *E. coli*-challenged groups had higher concentrations of TNF-α and S-IgA in jejunal mucosa than unchallenged birds (*p* < 0.001). Supplementation of ABPS increased the concentration of S-IgA in jejunal mucosa, but decreased the concentration of TNF-α in jejunal mucosa.

### 3.4. Response of NF-κB and EKR1/2 in the Jejunum

**Figure 2:**
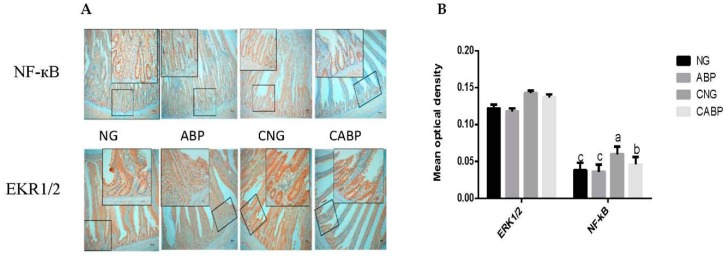
Response of NF-κB and EKR1/2 in the jejunum. (**A**) NF-κB and EKR1/2 immunostaining of mucosa (original magnification 100× and 400×). Only brown-colored staining was considered positive. (**B**) Density quantification of brown-colored staining. Significant interaction between *E. coli* challenge and ABPS was observed for NF-κB activation, but not for ERK1/2 activation (*p* = 0.055). Values are means ± SE, *n* = 6. Different superscripted lowercase letters within each group mean the values differ significantly (*p* < 0.05). NG: basal diet; ABP: supplemented with 500 mg ABPS additive per kg of diet; CNG: basal diet group with *E. coli* challenge; CABP: ABP group with *E. coli* challenge.

The effects of ABPS and *E. coli* challenge on NF-κB and ERK1/2 in yellow-feathered broilers were detected by immunohistochemical analysis (Figure 2). The interaction between *E. coli* challenge and ABPS was observed for NF-κB throughout the trial. The ABP group had a lower NF-κB response compared to other treatments (*p* < 0.001) (Figure 2A). Birds challenged with *E. coli* had higher (*p* < 0.001) responses of EKR1/2 compared with the saline group. However, no significant difference (*p* = 0.055) was observed in responses of EKR1/2 in the group supplemented with ABPS (Figure 2B).

### 3.5. The Change in Intestinal Mucosal Morphology

**Figure 3:**
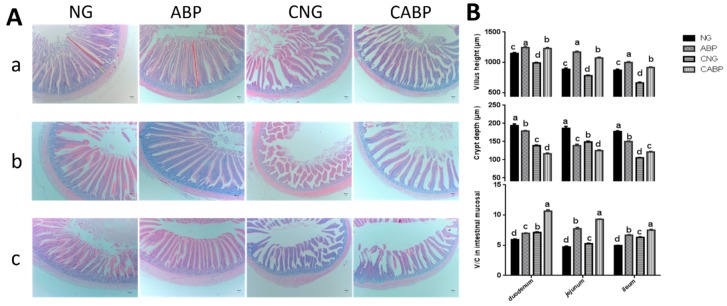
Effects of ABPS and *E. coli* challenge on intestinal mucosal morphology. (**A**) Intestinal (a: Duodenum; b: Jejunum; c: Ileum) mucosal morphology of 28-day-old yellow-feathered broilers was observed (40×). (**B**) The villi lengths, crypt depth, and the ratio of the villus length and crypt depth (V/C) were determined in intestinal samples. Values are means ± SE, n = 6. Different superscripted lowercase letters within each group mean the values differ significantly (*p* < 0.05). NG: basal diet; ABP: supplemented with 500 mg ABPS additive per kg of diet; CNG: basal diet group with *E. coli* challenge; CABP: ABP group with *E. coli* challenge.

The changes in intestinal mucosal morphologies for the duodenum, jejunum, and ileum were observed as shown in Figure 3, and the interaction effects between *E. coli* challenge and ABPS were observed for intestinal mucosal morphology throughout the trial. The ABP group had the highest intestinal villus height compared with other treatments (*p* < 0.001), while the CABP group had the highest villus height/crypt depth ratio (V/C) compared with other treatments (*p* < 0.001). On the other hand, the CNG group had the lowest intestinal crypt depth at the ileum compared with the other treatment groups (*p* < 0.001). The CABP group had the smallest intestinal crypt depth at the duodenum and jejunum compared with the other treatments (*p* < 0.001).

### 3.6. Cecal Microbial Community Structure

**Figure 4:**
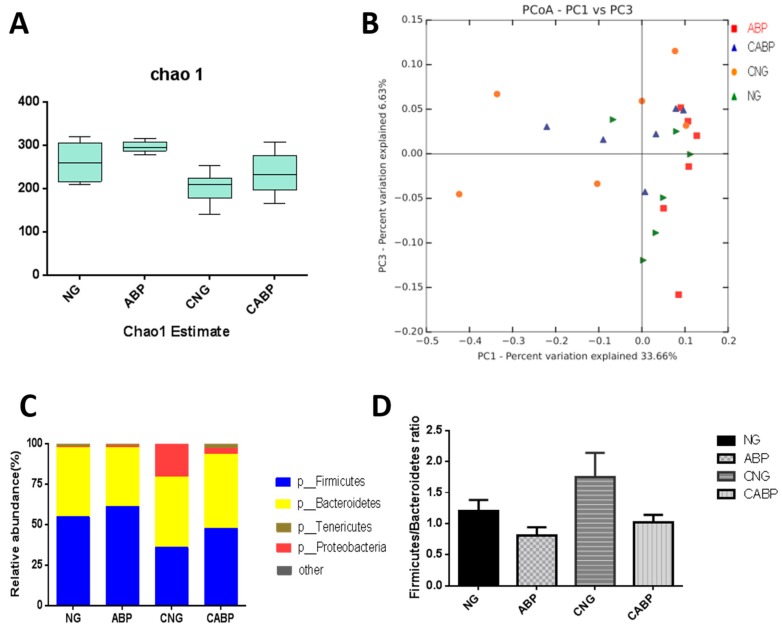
Effect of ABPS and *E. coli* challenge on the cecal microbiota. (**A**) Cecal taxa richness assessed by α-diversity analyses using the chao1 method. (**B**) Cecal microbial community β-diversity (unweighted Unifrac, *p* > 0.05), which was demonstrated using principal coordinate analysis (PCoA) of the unweighted Unifrac distance matrices. Microbial communities were clustered according to challenge (*E. coli*-challenged vs. saline); however, there was no obvious clustering according to diet supplementation (ABPS vs. non-ABPS). The first two principal coordinates (PC1 and PC3) are plotted for each bird. The percentage of dataset variability explained by each principal coordinate is shown in the axes’ titles. Each dot represents one bird and each group is denoted by a different color (green, NG; orange, CNG; blue, CABP; red, ABP). (**C**) Microbiota taxa composition at the phylum levels is shown, labeled according to phyla (p) where available *E. coli* was significant (*p* < 0.05), and more description is provided in the results section. (**D**) The *Firmicutes*/*Bacteroidetes* ratio is shown. For the ABPS and *E. coli* treatment, this was significant (*p* < 0.05), and more description is provided in the results section. NG: basal diet; ABP: supplemented with 500 mg ABPS additive per kg of diet; CNG: basal diet group with *E. coli* challenge; CABP: ABP group with *E. coli* challenge.

To determine the effect of ABPS and *E. coli* challenge on the baseline microbial community structure, the intestinal bacterial richness and diversity was determined by 16S rRNA analyses. To determine whether cecal taxa richness was altered by these treatments, we performed α-diversity analyses using the chao1 method (Figure 4A), which showed no interaction effect of *E. coli* challenge with ABPS throughout the trial period. α-diversity analyses demonstrated significant differences between *E. coli-*challenged and saline-receiving birds (*E. coli* 280.07 ± 16.18 vs. saline 218.89 ± 16.18; *p* = 0.015), but not between ABPS and non-ABPS birds (ABPS 266.42 ± 16.18 vs. non-ABPS 232.54 ± 16.18, *p* = 0.154), indicating that *E. coli* challenge reduced taxa richness in the cecal samples. Furthermore, microbial community diversity in the *E. coli*-challenged and non-*E. coli-*challenged groups was different, but not significantly (β-diversity (unweighted Unifrac); (*p* > 0.05)), and this was also demonstrated using principal coordinate analysis (PCoA) of the unweighted Unifrac distance matrices (Figure 4B). The PCoA plot showed that the microbial communities clustered according to challenge (*E. coli-*challenged vs. saline); however, there was no obvious clustering according to diet supplementation (ABPS vs. non-ABPS). The microbiota taxa composition in the cecum of birds is shown at the phylum level in Figure 4C,D and at the genus level in Table 4. As indicated, *Bacteroides* and *Firmicutes* were the dominant phyla in these samples. A significant and drastic increase in *Proteobacteria* and *Bacteroides* and a decrease in *Firmicutes* were observed in the *E. coli-*challenged groups; for example, *Proteobacteria* was more abundant in the *E. coli-*challenged birds (*E. coli* 11.92 ± 0.004 vs. saline, 0.63 ± 0.004; *p* = 0.028), but not between ABPS and non-ABPS supplemented groups (*p* > 0.05). The interaction effect of *E. coli* challenge with ABPS was not observed in the *Firmicutes*/*Bacteroidetes* ratio. The *E. coli* challenge treatment significantly reduced the *Firmicutes*/*Bacteroidetes* ratio in birds compared with the saline treatment (*E. coli* 0.92 ± 0.08 vs. saline 1.478 ± 0.08; *p* < 0.001), and also significant differences in the *Firmicutes*/*Bacteroidetes* ratio were observed between ABPS and non-ABPS birds (ABPS 1.39 ± 0.08 vs. non-ABPS 1.008 ± 0.08, *p* = 0.003). In addition, there was and *E. coli* challenge × ABPS interaction effect observed for some microbial species throughout the trial period. Subsequently, *E. coli*-challenged birds given the ABPS-supplemented diet (group CABP) had lower *E. coli* and *Enterococcus* contents than the birds in the CNG treatment group. Also, *Lachnospiraceae*, *Ruminococcaceae*, and some genera within the *Firmicutes* phylum were significantly more abundant in the groups given ABPS-supplemented diets.

## 4. Discussion

The results of the present study indicated that *E. coli* challenge decreased bird’s weight gain and feed intake, which is consistent with some previous studies [24,25]. Furthermore, the bird group treated with CABP had greater BW and ADG than in the CNG group, indicating the ability of ABPS to alleviate the negative effect from *E. coli* infection in birds. This observation is similar to the report of Guo et al. that ABPS supplementation had beneficial effects on the performance of weaned pigs challenged with *E. coli* [13]. On the other hand, serum glucose and uric acid levels were reduced in the birds fed by ABPS-supplemented diets. The glucose-lowering effect may be indicative of a role of ABPS in enhancing glucose disposal. Although the mechanisms by which ABPS decreases blood glucose concentrations are not clearly understood, the regulation of ABPS on insulin sensitivity may be involved in the response. A growing body of literature is supporting a role for ABPS supplementation in improving insulin resistance [26,27,28].

The present study showed that *E. coli* challenge significantly affected the birds’ serum biochemical parameters such as albumin and total protein. The *E. coli* challenge reduced serum total protein levels significantly, suggesting that *E. coli* K88 induced immunological stress that adversely affected the health of the birds. However, these changes might be associated with the impairment of functions, such as amino acid transport and protein synthesis, usually related to oxidative stress and toxicity [29,30]. In our study, the addition of ABPS in the diet increased the levels of serum total protein, albumin, and globulin in both *E. coli*-challenged and unchallenged birds. The increased serum concentration of globulin and albumin is an indication of enhanced immune system activity, regarded as the direct reference to the body immune function [31]. 

In regard to the circulating concentrations of the inflammatory indices, TNF-α and IL-4 were found to increase after *E. coli* challenge, which is consistent with the observations of Lee et al. and Liu et al. [32,33]. However, the birds fed ABPS had significantly lower concentrations of TNF-α and IL-4 than the birds fed the basal diet (NG) and the *E. coli*-challenged group (CNG), indicating that ABPS had an anti-inflammatory role. Moreover, ABPS played a significant role in suppressing the effects of *E. coli* infection in the CABP group by lowering the concentrations of TNF-α and IL-4 compared to the CNG group. Subsequently, the *E. coli*-challenged birds fed with ABPS (CABP) had the highest concentrations of S-IgA among these groups. This might be as a result of ABPS enhancing the production of S-IgA, which acts as the first line of defense against invading pathogens [34]. Many microbial pathogens make initial contact with their hosts at mucosal surfaces, especially in the alimentary tract. It has been reported that ABPS has anti-inflammatory properties and also improves body immune function, but the mechanism for its anti-inflammatory role in the animals still remains unclear [35,36]. NF-κB and MAPK signaling are important cell signaling pathways that regulate the production of cytokines and proinflammatory mediators such as IL-1, IL-6, TNF-α, and iNOS during the inflammatory response, although the cellular signaling pathways regulating inflammation are very complicated. In this study, the birds fed with ABPS showed lower expressions of NF-κB than the birds fed either with the basal diet or receiving *E. coli* challenge. This result is consistent with ABPS relieving immune stress through the reduced expression levels of NF-κB protein in piglet jejunum epithelial cells under the immunological stress of lipopolysaccharides, as observed by Wang et al. [37]. 

In this study, birds fed with ABPS had longer intestinal villi compared with the other birds. The intestinal villus and crypt depth of the birds were decreased significantly under the conditions of *E. coli* infection in the challenge group. The challenge of *E. coli* K88 led to villus atrophy and intestinal morphology disruption [38]. It is known that the intact morphology of the mucosa in the duodenum, jejunum, and ileum is one of the most important indications of intestinal health as well as digestive and absorptive capacity in poultry [39,40]. Hence, our results indicated that dietary supplementation with ABPS played a beneficial role in improving the intestinal morphology of the birds, including the *E. coli*-challenged ones. 

In this study, *E. coli-*challenged birds had a lower proportion of *Firmicutes* and an increase in the abundance of *Proteobacteria*. The taxonomic profiling demonstrated that ABPS treatment could increase the level of *Firmicutes* and reduce the level of *Proteobacteria* and *Bacteroidetes*, modulating the gut composition of birds to levels similar to those of the unchallenged birds. Moreover, Peter et al. and Koliada et al. found that the content of *Firmicutes* and the *Firmicutes*/*Bacteroidetes* ratio were gradually increased while the content of *Bacteroidetes* was decreased with increasing body mass index (BMI) in humans [41,42]. In line with this, our results showed that ABPS supplementation in the diet of *E. coli*-challenged birds could increase their BW and ADG, unlike the challenged birds with no supplement; hence, regarding the increase in the *Firmicutes*/*Bacteroidetes* ratio, a possible explanation for our findings is that *Firmicutes* are more effective as an energy source than *Bacteroidetes*, thus promoting more efficient absorption of carbohydrates and subsequent weight gain [43,44]. *Proteobacteria* is a major phylum of bacteria which includes a wide variety of pathogens, such as *Escherichia*, *Salmonella*, *Vibrio*, and many other notable genera [45]. In the current study, *E. coli*-challenged birds on the ABPS-supplemented diet had lower *Escherichia coli* and *Enterococcus* contents than the *E. coli-*treated birds fed a basal diet. Furthermore, in recent decades, *Enterococcus faecalis* has also emerged as a pathogen, especially in nosocomial infections, and this suggests that ABPS has the tendency to selectively reduce the abundance of disease-causing microorganisms [46]. The study by Xie et al. reported that ABPS increased the numbers of *Lactobacilli* and *Bifidobacterium* and reduced the numbers of *E. coli* in weaned piglets. Similarly, the present study showed that birds receiving the ABPS diet had an abundance of *Ruminococcaceae* and *Lachnospiraceae* [47]*.* The *Ruminococcaceae*, which is a producer of acetate and butyrate, is involved in the first step of microbiome-associated carbohydrate metabolism, degrading several types of polysaccharides in the intestinal tract [48]. Also, *Lachnospiraceae* are the producers of butyrate and have been linked to protection from colon cancer in humans [49,50]. The higher abundance of *Ruminococcaceae* and *Lachnospiraceae* in the ABPS-supplemented group means that short-chain fatty acid (SCFA) production was increased. Moreover, inhibition of pathogen replication has been shown to be mediated by low-molecular-weight substances, primarily SCFAs [51]. These SCFAs exert therapeutic effects on some human and experimental animal diseases [52,53]. The intestine harbors a complex microbial community that plays a key role in nutrition and health [54]. These results indicated that ABPS, apart from combating pathogens, may promote a more symbiotic intestinal microflora favoring the host when challenged with an enteric pathogen such as *E. coli.*

## 5. Conclusions

The results of the present study demonstrated that enteric disease challenge with *E. coli* K88 decreases growth performance in broilers, and dietary supplementation of ABPS can serve as an effective prophylactic treatment to alleviate this growth suppression. Dietary ABPS supplementation for birds could enhance the initiation of the immunological response to *E. coli* infection by mediating the release of immunoglobulins, such as S-IgA, and improving the cecal microflora. These actions finally promote the general health and growth performance of birds.

## Figures and Tables

**Table 1:** Composition of the basal diet.

Item	Basal Diet (0 to 4 weeks)
Ingredient, %	
Ground yellow maize	56.65
Soybean meal	36
Soybean oil	3.0
Dicalcium phosphate	1.8
Limestone	1.0
NaCl	0.3
DL-Met ^3^	0.1
Choline chloride	0.15
Premix ^1^	1.0
Nutrient level ^2^	
ME ^3^, MJ/kg	12.22
CP ^3^, %	20.10
Lys, %	1.02
Met, %	0.42
Cys,%	0.32
Ca,%	1.11
Available P, %	0.54

^1^ Supplied, per kilogram of diet: Cu, 10 mg; Fe, 90 mg; Mn, 90 mg; Zn, 50 mg; Se, 0.2 mg; I, 0.4 mg; Co, 0.4 mg; vitamin A, 5000 IU; cholecalciferol, 500 IU; vitamin E, 10 IU; riboflavin, 6.0 mg; pantothenic acid, 12 mg; niacin, 35 mg; cobalamin, 10 µg; biotin, 0.8 mg; folic acid, 0.8 mg; thiamine, 1.5 mg; and pyridoxine, 1.5 mg. ^2^ Based on the Nutrient Requirements of yellow broilers (China, NY/T 33-2014) and the Nutrient Requirements of Broilers (NRC, 1994). ^3^ DL-Met: DL-Methionine; ME: Metabolizable Energy; CP: Crude Protein.

**Table 2:** Effects of ABPS and *E. coli* challenge on growth performance of yellow-feathered broilers.

Item	*−E. coli*		*+E. coli*		SEM	*p*-Value		
	NG	ABP	CNG	CABP		ABPS	*E. coli*	ABPS * *E. coli*
BW (g)	635.17 ^a,b^	651.1 ^a^	579.5 ^c^	627.33 ^b^	6.49	<0.001	<0.001	0.038
ADG (g)	21.6 ^a,b^	22.16 ^a^	19.61 ^c^	21.32 ^b^	0.13	<0.001	<0.001	0.038
ADFI (g)	44.83	44.57	42.58	44.55	0.39	0.298	0.168	0.172
FCR (g:g) ^1^	2.08	2.01	2.17	2.09	0.02	0.002	<0.001	0.658
Mortality (%)	5.00	3.33	8.33	5.00	1.03	0.242	0.242	0.692

BW: body weight; ADG: average daily body weight gain; ADFI: average daily feed intake; FCR: feed/gain ratio; ^a,b^ within a row, values with different superscripts differ significantly (*p* < 0.05); Each mean represents 6 replicates. NG: basal diet; ABP: supplemented with 500 mg ABPS additive per kg of diet; CNG: basal diet group with *E. coli* challenge; CABP: ABP group with *E. coli* challenge; ^1^ ABPS and *E. coli* was significant (*p* < 0.05), and more description is provided in the results section.

**Table 3:** Effects of ABPS and *E. coli* challenge on serum biochemical indices of yellow-feathered broilers.

Item	*−E. coli*		*+E. coli*		SEM	*p*-Value		
	NG	ABP	CNG	CABP		ABPS	*E. coli*	ABPS * *E. coli*
ALB (g/L)	13.98	14.66	13.23	14.18	0.24	0.104	0.198	0.763
TP ^1^ (g/L)	35.28	42.42	32.15	40.75	1.01	<0.001	0.008	0.375
GLB ^2^ (g/L)	21.28	27.77	19.31	26.57	0.91	<0.001	0.091	0.669
GLU (mmol/L) ^3^	14.49	13.15	13.66	12.39	0.17	<0.001	<0.001	0.744
UA(μmol/L) ^4^	273.19	242.56	295.55	279.49	4.35	<0.001	<0.001	0.059

GLU: glucose; TP: total protein; ALB: albumin; GLB: globulin; UA: uric acid; ^a,b^ within a row, values with different superscripts differ significantly (*p* < 0.05); Each mean represents 6 replicates. NG: basal diet; ABP: supplemented with 500 mg ABPS additive per kg of diet; CNG: basal diet group with *E. coli* challenge; CABP: ABP group with *E. coli* challenge; ^1,2^ ABPS was significant (*p* < 0.05), and more description is provided in the results section; ^3,4^ ABPS and *E. coli* was significant (*p* < 0.05), and more description is provided in the results section.

**Table 4:** Effects of ABPS and *E. coli* challenge on some different genera in yellow-feathered broilers.

Taxonomy Units	*-E. coli* % Abundance		*+E. coli* % Abundance		^ab^*p* Value			SEM
	NG	ABP	CNG	CABP	ABPS	*E. coli*	ABPS * *E. coli*	
*p__Firmicutes; c__Clostridia; o__Clostridiales; f__Lachnospiraceae; g__Coprococcus_1; s__unidentified(OTU 2)*	15.87 ^ab^	21.08 ^a^	9.31 ^b^	13.20 ^b^	0.849	0.007	0.038	0.015
*p__Bacteroidetes; c__Bacteroidia; o__Bacteroidales; f__Bacteroidaceae; g__Bacteroides; s__unidentified (OTU 6)*	2.13	3.14	4.31	4.81	0.056	0.001	0.474	0.003
*p__Bacteroidetes; c__Bacteroidia; o__Bacteroidales; f__Bacteroidaceae; g__Bacteroides(OTU11)*	2.97	4.28	3.98	5.16	0.015	0.003	0.848	0.003
*p__Proteobacteria;c__Gammaproteobacteria; o__Enterobacteriales;f__Enterobacteriaceae; g__Escherichia-Shigella; s__Escherichia_coli(OTU 4)*	0.19 ^b^	0.85 ^b^	12.34 ^a^	3.41 ^b^	0.055	0.004	0.031	0.016
*p__Firmicutes; c__Bacilli; o__Lactobacillales; f__Enterococcaceae; g__Enterococcus; s__unidentified(OTU 27)*	0.20 ^b^	0.01 ^b^	3.15 ^a^	0.04 ^b^	0.001	0.001	0.001	0.004
*p__Firmicutes; c__Clostridia; o__Clostridiales; f__Lachnospiraceae; g__Eisenbergiella; s__unidentified(OTU 32)*	0.58	1.31	0.35	0.21	0.003	0.003	0.027	0.001
*p__Firmicutes; c__Clostridia; o__Clostridiales; f__Ruminococcaceae; g__Ruminococcaceae_UCG-014; s__unidentified(OTU201)*	0.32 ^b^	1.17 ^a^	0.31 ^b^	0.34 ^b^	0.037	0.005	0.005	0.001
*p__Firmicutes; c__Clostridia; o__Clostridiales; f__Ruminococcaceae(OTU 56)*	0.08	0.51	0.05	0.31	0.028	0.522	0.294	0.0004
*p__Firmicutes; c__Clostridia; o__Clostridiales; f__Ruminococcaceae; g__Ruminococcaceae_UCG-014; s__unidentified(OTU63)*	0.00	0.43	0.00	0.11	0.07	0.189	0.271	0.001

NG: basal diet; ABP: supplemented with 500 mg ABPS additive per kg of diet; CNG: basal diet group with *E. coli* challenge; CABP: ABP group with *E. coli* challenge; ^a^ Overall difference between groups was determined using ANOVA. ^b^ Differences between groups were determined by Duncan’s multiple comparison test. ^a,b^ Within a row, values with different superscripted letters differ significantly (*p* < 0.05).
